# Supplementary material for: The mediating effects of public genomic knowledge in precision medicine implementation: A structural equation model approach
Source: PLoS One. 2020 Oct 14;15(10):e0240585. doi: 10.1371/journal.pone.0240585 (PMC7556538; doi:10.1371/journal.pone.0240585)
Supplement: Attachment — Submitted filename: PONE-D-19-33144.docx [file pone.0240585.s001.docx]

This manuscript presents how characteristics of omics biomarker influence precision medicine implementation through public genomic awareness, which is interesting. However, there are some problems:

1. The manuscript did follow the regular scientific writing format. For example, we do not use “research methodology” headings, we use “methods”; we do not use “statistical considerations” headings, we use “data analysis”. The authors did not have participants and procedure sessions.

2. The authors did not provide information about normality and outliers.

3. No information about the model fits of the SEM model.
