# Supplementary material for: The mediating effects of public genomic knowledge in precision medicine implementation: A structural equation model approach
Source: PLoS One. 2020 Oct 14;15(10):e0240585. doi: 10.1371/journal.pone.0240585 (PMC7556538; doi:10.1371/journal.pone.0240585)
Supplement: Attachment — Submitted filename: June Author response to Reviewer Comments.docx [file pone.0240585.s002.docx]

**Authors’ Response to Reviewer Comments**

**Manuscript Title: The mediating effects of public genomic knowledge in precision medicine implementation: structural equation model approach**

| **Reviewer Comments** | **Authors’ Response** | **Sections where changes are made in Revised Manuscript** |
| --- | --- | --- |
| Reviewer # 1 |  |  |
| 1. The manuscript did follow the regular scientific writing format. For example, we do not use “research methodology” headings, we use “methods”; we do not use “statistical considerations” headings, we use “data analysis”. The authors did not have participants and procedure sessions. | **Answer:**  “Research methodology” subtitle changed to read “Methods” as suggested;  “Statistical considerations” subtitle changed to read “Data analysis”  Although presented implicitly in a narrative form in the previous draft, the “participants and procedure” section is now more explicitly presented as a subtitle in the revised draft manuscript. | Line **183**  Line **253**  From lines **184** to **218** |
| 2. The authors did not provide information about normality and outliers. | **Answer:**  Information about normality and outliers for the data has now been provided. | Lines **306** t0 **316** |
| 3. No information about the model fits of the SEM model. | **Answer:**  Information about the model fits of the SEM model has now been provided. | Lines **241** to **244** |
| **Reviewer # 2** |  |  |
| 1. In the abstract, "Results from the individual mediation paths ‘a’ and ‘b’ however, showed that these effects were negative". Because your model is not presented in the abstract, my suggestion is to explain what ‘a’ and ‘b’ referred to. | **Answer:**  The Abstract now contains a brief statement explaining what ‘a’ and ‘b’ refer to. | Lines **26** to **30** |
| 2. In order to understand the quantitative relationship between variables more clearly, is it possible to label the values in the model diagram? | **Answer:**  The model diagram now has the mediation coefficients labelled as suggested | Figure 5 |
| 3. The interpretation of the hypothetical results is slightly weak; it is recommended to further explore the results of the hypotheses. | **Answer:**  We have now added sections on our hypotheses interpretation section to further explore the results of the hypotheses and show how findings tie up with existing literature | Lines **447** t0 **485**  Lines **1509** to **520**  Lines **542** t0 **563** |
| **Reviewer # 3** |  |  |
| 1. A major issue with the study is the exclusion of other significant relevant factors associated with implementation outcomes. It is often seen that two factors are significantly associated, but when including other factors associated with the dependent variable, the relationship diminished or disappeared. A more though lit review needs to be provided and including other important variables related to implementation outcomes in the model is necessary | **Answer:**  We believe this is a genuine concern in that the existence of unmeasured variables reflect a violation of important assumptions in path analysis. In response, and in recognizing that measuring all potentially impactful variables for the model would be impossible to achieve – as has been pointed out in related literature - we considered the question of the degree to which the unavoidable unmeasured variables potentially biased estimates of our path coefficients and provided a basis for alternative explanations of our findings. Consequently, in the revised draft, we have focused on why the presented model would likely be the most appropriate and argued out the plausibility of an alternative model based on any omitted unmeasured variables. | Lines **542** t0 **563** |
| 2. Relatedly, the authors discussed factors at different levels (e.g. innovation-level factors, individual-level factors, and organizational factors, etc.), but failed to include such factors in the quantitative model. Excluding such factors will bias the model results. Accordingly, multi-level structural equation modeling should be considered. | **Answer:**  In addressing this concern, we have added a section on multilevel SEM in the revised draft. We have shown that even though factors hypothesized to affect PM implementation in our study may seem to be premised on a multilevel framework , based on the available data, a multi-level structural equation modeling would not have been applicable. This is in agreement with other published works (e.g. Sophia Rabe-Hesketh, Anders Skrondal and Xiaohui Zheng, 2007(*Multilevel Structural Equation Modeling)* and Bengt O Muthen, 2011 (*Mean and covariance structure analysis of hierarchical data*)) that indicate that whereas in conventional structural equation models, all latent variables and indicators vary between units and are assumed to be independent across units, the latter assumption is violated in multilevel settings where units are nested in clusters, leading to within-cluster dependence. This is further addressed in the revised manuscript | Lines **286** t0 **294** |
| 3. The conclusion section failed to fully explain the cause for the relationship identified through the model. How is it tied to the literature? What are the other important factors? | **Answer:**  This is indeed related to Reviewer #2’s Observation #3. We have accordingly added sections on our hypotheses interpretation section to further explore the results of the hypotheses and show how findings tie up with existing literature. More specifically, we considered the question of the degree to which the unmeasured variables could have potentially biased estimates of path coefficients and provided a basis for alternative explanations of our findings. We showed how, on the basis of prior knowledge, research design used, and empirical analysis of the data used in the study, alternative models to the one presented had to be ruled out. | Lines **543** t0 **564** |
| 4. Participants’ demographic information should be included in the model. In particular, is there any differentiated effects across subgroups, e.g., gender and age? Moderation effects need to be examined in the structural equation modeling. | **Answer:**  We did not do an analysis by gender and age because we did not have good representation of those groups. This is because we regrettably realized that our data does not meet important condition/assumptions for a moderated mediation: sub-groups must have similar group sizes because path coefficients depend upon subgroup sizes. Due to this consideration, we did not consider moderation as suggested. We have consequently noted this as a study limitation in our study. | Lines **588** - **590** |
| 5. The authors failed to explain the high attrition rate in the study (i.e. only 270 out of 442 participants completed the survey), and this may bias the study results. For example, were the participants who completed the surveys representative of the population that the authors intended to measure? | **Answer:**  Online means of data collection are prone to increased risk of survey attrition—participants dropping out. To address this risk and possibly curb the potential havoc such participant attrition may have on our study findings, we set crucial question settings on the online survey tool to ‘compulsory’. This implied that participants had to answer the first survey question to proceed to the next one and all questions had to be completed before successfully submitting the survey form at the finish line. However, the survey platform still recorded any attempt to participate, completed or not. This measure meant that attempt by quitters to participate was registered. Unfortunately, this measure did not discriminate between genuine quitters and subsequent attempters. To eliminate possibility of a bias due to survey attrition or attrition affecting study findings and thus having a negative statistical implication on our model, we only used data from participants who actually completed the survey for all statistical analyses. | Lines **206** t0 **218** |
| 6. The authors should pay more attention to the details of the manuscript. For example, there were inconsistent spacing thought the manuscript, e.g. reference 13. There were also some missing texts, e.g. page 11 “systemic amyloidosis ().” | **Answer:**  We have proof-read the manuscripts and attended to all the formatting issues including the referencing | Line **76** |
