# Supplementary material for: The mediating effects of public genomic knowledge in precision medicine implementation: A structural equation model approach
Source: PLoS One. 2020 Oct 14;15(10):e0240585. doi: 10.1371/journal.pone.0240585 (PMC7556538; doi:10.1371/journal.pone.0240585)
Supplement: Attachment — Submitted filename: CJ September Author response to PLOS1 Reviewer Comments.docx [file pone.0240585.s003.docx]

Authors’ Response to 2^nd^ Round Reviewer Comments

**Manuscript Title:** The mediating effects of public genomic knowledge in precision medicine implementation: structural equation model approach

| **Reviewer Comments** | **Authors’ Response** | **Sections where changes are made in Revised Manuscript** |
| --- | --- | --- |
| Reviewer # 4 |  |  |
| 1.Need to report the average age , education background, family economic status and other basic demographic information about the subjects. | We have included Fig 3 to show basic demographic details of study participants. | Lines **313** to **318** |
| 2. The reliability and validity of measurement tools need to be reported in manuscripts. | Information about measurement consistency and validity, together with the measurement model goodness of fit indices have now been provided. | Lines **242** to **253** |
| 3. The measurement consistency coefficient of the items must to be reported. | An omega reliability coefficient of 0.95 has been provided in the revised manuscript to indicate measurement consistency of the items in the tool | Lines **248** |
| 4. Please introduce the software and version for data analysis. | We have made revisions to include brief statements that introduce both “likert” and “lavaan” R packages, while package versions have also been indicated. | Lines **267** to **273** |
